# Plasma kallistatin in critically ill patients with severe sepsis and septic shock

**DOI:** 10.1371/journal.pone.0178387

**Published:** 2017-05-24

**Authors:** Wei-Chieh Lin, Chang-Wen Chen, Lee Chao, Julie Chao, Yee-Shin Lin

**Affiliations:** 1 Department of Internal Medicine, National Cheng Kung University Hospital, College of Medicine, National Cheng Kung University, Tainan, Taiwan; 2 Department of Biochemistry and Molecular Biology, Medical University of South Carolina, Charleston, South Carolina, United States of America; 3 Department of Microbiology and Immunology, College of Medicine, National Cheng Kung University, Tainan, Taiwan; 4 Center of Infectious Disease and Signaling Research, National Cheng Kung University, Tainan, Taiwan; National Yang-Ming University, TAIWAN

## Abstract

Kallistatin, an endogenous serine proteinase inhibitor, is protective against sepsis in animal models. The aim of this study was to determine the plasma concentration of kallistatin in intensive care unit (ICU) patients with severe sepsis and septic shock and to determine their potential correlation with disease severity and outcomes. We enrolled 86 ICU patients with severe sepsis and septic shock. Their plasma concentrations of kallistatin, kallikrein, tumor necrosis factor (TNF)-α, interleukin (IL)-1β, IL-6, and IL-8 were measured by enzyme-linked immunosorbent assay. The association of kallistatin levels with disease severity and patient outcomes was evaluated. The relationship between kallistatin and other biomarkers was also analyzed. Plasma kallistatin levels on day 1 of ICU admission were lower in patients with septic shock compared with patients with severe sepsis (p = 0.004). Twenty-nine patients who died in the hospital had significantly lower day 1 kallistatin levels than patients who survived (p = 0.031). Using the optimal cutoff value (4 μg/ml) of day 1 plasma kallistatin determined by receiver operating characteristic curves for 60-day mortality, we found that high kallistatin levels were associated with a preferable 60-day survival (p = 0.012) by Kaplan-Meier analysis and lower Sequential Organ Failure Assessment (SOFA) scores over the first 5 days in the ICU (p = 0.001). High kallistatin levels were also independently associated with a decreased risk of septic shock, the development of acute respiratory distress syndrome, and positive blood cultures. In addition, there were inverse correlations between day 1 kallistatin levels and the levels of TNF-α, IL-1β, IL-6, and C-reactive protein, and SOFA scores on day 1. Our results indicate that during severe sepsis and septic shock, a decrease in plasma concentrations of kallistatin reflects increased severity and poorer outcome of disease.

## Introduction

Sepsis is a major cause of death in the intensive care unit (ICU). Between 11% and 15% of patients admitted to ICUs have or will develop severe sepsis, and the mortality rate for these patients varies between 30% and 60% [1–3]. Sepsis is characterized by a generalized overwhelming inflammatory process and subsequent development of multiple organ dysfunction, potentially leading to death. Despite recent advances in its early diagnosis, the early administration of antibiotics and fluids, hemodynamic optimization, and better technological support of organ function, treatment of sepsis remains exclusively expectant and supportive [4]. Adjunctive treatments targeting perturbations in the innate immune response and coagulation cascade have been evaluated, but none was proven beneficial [4]. However, pharmacological agents that interfere with the effects of inflammation during sepsis are still of intense clinical interest.

Kallistatin, a human serine proteinase inhibitor, was first identified as a tissue kallikrein-binding protein and functions as an endogenous tissue kallikrein inhibitor [5–7]. Kallistatin exhibits pleiotropic effects including anti-angiogenesis, anti-oxidation, anti-apoptosis, and anti-inflammation in animal models and cultured cells, thereby protecting against vascular and organ damage, independent of its interaction with tissue kallikrein [8–12]. Transgenic mice overexpressing kallistatin were more resistant to lipopolysaccharides (LPS)-induced lethality than the control group [13]. Kallistatin gene transfer also attenuated inflammation and liver damage and improved survival in group A streptococcus-infected mice [14]. Recent studies demonstrated that mice treated with human kallistatin protein prior to or following cecal ligation and puncture or LPS-induced sepsis showed attenuated organ injury, inflammation and apoptosis, enhanced bacterial clearance, and improved survival [15, 16]. We previously showed that kallistatin gene delivery or kallistatin protein administration ameliorated acute lung injury in LPS-treated mice [17]. Furthermore, studies in animal models of myocardial ischemia-reperfusion injury, chronic myocardial infarction and salt-induced renal injury reported that kallistatin gene delivery was protective against organ injury, inflammation, apoptosis, and oxidative stress, whereas kallistatin depletion by antibody injection exacerbated cardiovascular and renal damage and inflammation in hypertensive rats [10, 12, 18, 19]. In human studies, significantly reduced kallistatin levels were reported in plasma samples from patients with liver disease and sepsis [5]. Our previous reports also showed that lower plasma kallistatin levels were associated with increased mortality and disease severity in patients with community-acquired pneumonia (CAP) [20]; a lower ratio of kallistatin to total protein in the bronchoalveolar lavage fluid was associated with higher neutrophil counts in the bronchoalveolar lavage fluid and increased mortality in patients with sepsis-induced acute respiratory distress syndrome (ARDS) [17]. However, studies investigating the clinical value of plasma kallistatin as a biomarker to assess outcomes of patients with severe sepsis and septic shock remain to be elucidated.

Given the potential therapeutic benefit of kallistatin in sepsis, it is reasonable to monitor kallistatin levels in critically ill patients with severe sepsis and septic shock. The aim of this study was to assess the association of plasma kallistatin with disease severity and outcomes, and the relationship between kallistatin and other biomarkers in patients with severe sepsis and septic shock.

## Materials and methods

### Study design and setting

This prospective observational study included patients admitted to the medical ICU in National Cheng Kung University Hospital, a tertiary referral center in southern Taiwan. The institutional review board of the National Cheng Kung University Hospital approved the study (approval number ER-99-32). Written informed consent was obtained from patients or, if not possible, from their legal representatives.

### Study population

Patients with severe sepsis and septic shock were included in the study between April 2010 and December 2010. Exclusion criteria were pregnancy, human immunodeficiency virus infection, immunosuppressive treatment, and tuberculosis. Severe sepsis and septic shock were defined according to the criteria of the International Sepsis Definitions Conference [21]. Patients were also excluded based on refusal to provide informed consent, prior hospitalization within 14 days before admission, terminal illness receiving palliative treatment, and delayed ICU admission for more than 48 hours. All patients received treatment based on international guidelines for the management of sepsis and septic shock [21].

Blood cultures were performed before the administration of empirical antibiotics once infection was suspected. Positive blood cultures were defined as blood cultures positive for any pathogen other than coagulase-negative *Staphylococci* after alcohol sterilization. Coagulase negative *Staphylococci* were considered to be unlikely causes of sepsis unless present as the sole isolate in multiple blood cultures.

### Data collection

Clinical data such as age, sex, chronic diseases (diabetes, liver cirrhosis, chronic kidney disease, chronic heart disease, chronic respiratory disease, malignancy), primary sites of infection, microorganisms, complications (septic shock, ARDS, need for mechanical ventilation), clinical variables (blood pressure, body temperature, respiratory rate, heart rate), laboratory data (blood cell count, biochemical assays and arterial blood gas), and outcome data (length of ICU and hospital stay, duration of mechanical ventilation, hospital mortality) were obtained. The Acute Physiology and Chronic Health Evaluation II (APACHE II) score and quick Sequential Organ Failure Assessment (qSOFA) score on day 1 and SOFA score on days 1, 3, and 5 of ICU admission were calculated. Appropriate empirical antibiotics were defined according to previous definitions as at least one effective antibiotic included in the antimicrobial treatment within the first 24 h after diagnosis of sepsis. An effective antibiotic was a drug with *in vitro* activity to a susceptible isolated pathogenic organism(s). For patients without isolated pathogenic organisms, administered antibiotics consistent with internationally accepted norms for empirical therapy based on the patients’ local community and nosocomial flora were designated as appropriate. The dose and pattern of treatment were provided according to current medical standards.

### Plasma biomarker, routine blood cell count and biochemistry measurements

Blood samples were collected on days 1, 3, and 5 of ICU admission in heparinized tubes and centrifuged for 10 minutes at 3,000 ×*g*. Then, plasma aliquots were stored at −80°C until analysis. Levels of kallistatin, kallikrein, tumor necrosis factor (TNF)-α, interleukin (IL)-1β, IL-6 and IL-8 were measured in duplicate using enzyme-linked immunosorbent assay kits (R&D Systems, Minneapolis, MN, USA). Standard blood analyses, including blood cell count and biochemistry, were performed at the Clinical Central Laboratory, National Cheng Kung University Hospital, as part of routine analyses. Four of 86 (4.7%) samples from day 3 sampling and 12 samples (14%) from day 5 were missing because of disrupted sampling after the transfer of patients to wards or because of death.

### Study outcomes

Our primary outcome measure was in-hospital mortality. Secondary outcome measures were 60-day mortality and an association between kallistatin levels and disease severity. We also compared kallistatin levels on days 1, 3, and 5 in nonshock patients versus patients with septic shock, non-ARDS versus ARDS, and patients with negative blood culture versus positive blood culture. Patients were followed until death or 60 days after ICU admission.

### Statistical analysis

Kolmogorov-Smirnov and Shapiro-Wilk tests were performed to examine the normality of all variables. Continuous variables were reported as the mean ± standard deviation (SD), or median (interquartile range (IQR)). Categorical variables were expressed as frequencies (proportions). Continuous variables were compared by Student’s *t*-test or the Mann—Whitney *U*-test (for skewed data); categorical variables were compared using the chi-squared test or Fisher’s exact test, as appropriate. Levels of significance are expressed as p values; a two-sided p < 0.05 was considered statistically significant. Spearman’s non-parametric correlation coefficient (ρ) was used to calculate correlations between kallistatin and other investigated biomarkers and severity scores. Area under the curve (AUC) and receiver operating characteristic (ROC) curves were reported for day 1 biomarkers for 60-day mortality. The optimal cutoff was identified as the value corresponding to the highest sum of sensitivity and specificity (Youden index). Kaplan-Meier curves were generated for kallistatin to evaluate the ability to discriminate between patients who survived and those who died at 60 days after ICU admission. Differences between the Kaplan-Meier curves were assessed using the log-rank test. The prognostic value of biomarkers for 60-day mortality was assessed using the univariate Cox model. For multivariable analysis, the Cox proportional hazards regression model was used with adjustment for age, sex, septic shock, ARDS, SOFA score, and APACHE II score. Results are presented as the adjusted hazard ratios (HR) with 95% confidence intervals (CIs). A univariate logistic regression model was used to determine the predictive value of biomarkers for septic shock, ARDS, and positive blood culture. Variables that were significant (i.e. p < 0.05) on univariate analysis were entered into the backward multivariable analysis. The results are shown as the adjusted odds ratios (ORs) and 95% CIs. A mixed model was used to compare SOFA scores between groups stratified by high or low kallistatin levels according to the determined optimal cutoff value. Statistical analyses were performed using a statistical software package (PASW for Windows, version 20; SPSS Inc., Chicago, IL, USA) and GraphPad Prism 6.0 software (GraphPad Inc., La Jolla, CA, USA).

## Results

### Demographic and clinical characteristics of study cohort

**Fig 1:**
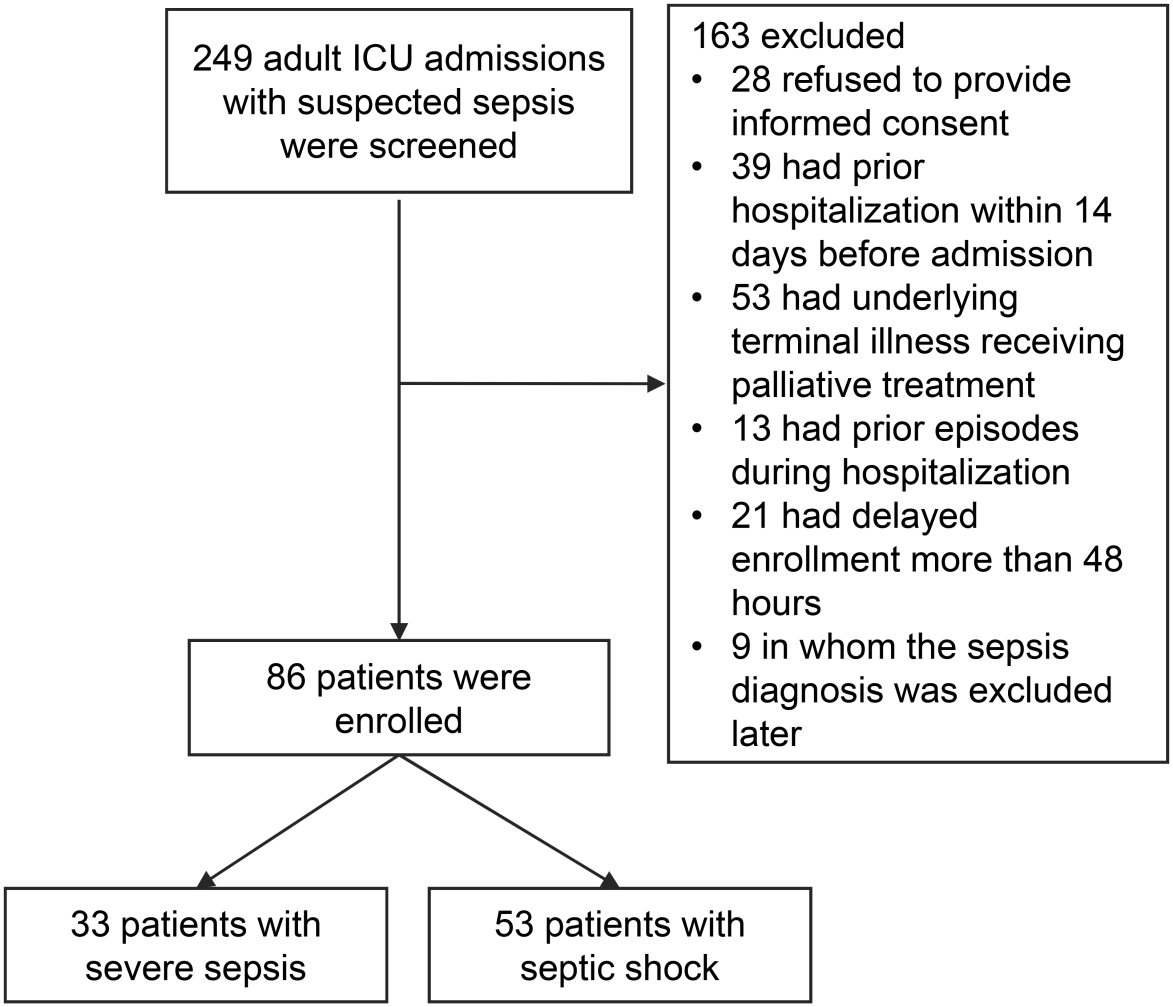
Flow chart of patient inclusion and exclusion.

Of 249 patients admitted to ICUs with suspected sepsis, 28 patients were screened but refused to provide informed consent, 39 had prior hospitalization within 14 days before admission, 53 had an underlying terminal illness receiving palliative treatment, 13 had prior episodes during hospitalization, 21 had delayed enrollment for more than 48 hours, and 9 had their diagnosis of sepsis excluded later; therefore, 86 patients were included in this study. Among these, 53 patients were diagnosed with septic shock and 33 patients with severe sepsis without shock (Fig 1). The demographic data and clinical variables are summarized in Table 1. The overall hospital mortality in the cohort was 33.7%. Survivors and nonsurvivors were similar in terms of age, sex, primary infection site, comorbidities, frequency of ARDS, white blood cell count at ICU admission, need for invasive mechanical ventilation, positive blood cultures, appropriateness of antibiotic treatment, and length of hospital stay. Compared with survivors, more nonsurvivors had septic shock, higher APACHE II, qSOFA and SOFA scores, longer length of ICU stay and duration of mechanical ventilation. Twenty-three patients (26.7%) had positive blood culture, and the corresponding microorganisms are shown in Table 2.

**Table 1:** Comparisons of characteristics between survivors and nonsurvivors of severe sepsis and septic shock.

Variables	Survivors (n = 57)	Nonsurvivors (n = 29)	p value
Age, years	69.6 ± 16.4	74.4 ± 9.6	0.091
Sex, male, n (%)	40 (70%)	17 (59%)	0.406
Pneumonia/extrapulmonary sepsis, n (%)	40 (70%)	19 (66%)	0.846
Comorbidities, n (%)			
Diabetes	22 (39%)	9 (31%)	0.651
Chronic kidney disease	13 (23%)	6 (21%)	1.000
Chronic respiratory disease	14 (25%)	9 (31%)	0.701
Chronic heart disease	19 (33%)	5 (17%)	0.187
Liver cirrhosis	10 (18%)	3 (10%)	0.529
Malignancy	2 (4%)	1 (3%)	1.000
ARDS, n (%)	7 (12%)	9 (31%)	0.069
Invasive mechanical ventilation, n (%)	54 (95%)	29 (100%)	0.548
Septic shock, n (%)	30 (53%)	23 (79%)	0.030
Positive blood cultures, n (%)	13 (23%)	10 (35%)	0.369
Appropriate antibiotic treatment	45 (79%)	23 (79%)	1.000
WBC (× 10^3^/μl)	14.0 ± 7.8	13.7 ± 7.7	0.870
APACHE II score, points	23.8 ± 6.4	29.6 ± 8.1	< 0.001
qSOFA score, points	2.0 (2.0–3.0)	3 (2.0–3.0)	0.013
SOFA score, points			
Day 1	8.4 ± 2.9	11.7 ± 3.7	< 0.001
Day 3	7.0 (4.5–9.0)	10.5 (8.0–15.8)	< 0.001
Day 5	5.0 (4.0–7.0)	11.0 (6.0–14.0)	< 0.001
ICU stay, days	5.0 (4.0–10.0)	10.0 (4.5–17.5)	0.029
Hospital stay, days	17.0 (12.0–34.0)	15.0 (6.5–25.5)	0.113
Mechanical ventilation, days	5.0 (3.0–8.5)	10.0 (5.0–14.5)	0.022

Data are presented as the mean ± standard deviation or median (interquartile range) unless otherwise stated. ARDS, acute respiratory distress syndrome; ICU, intensive care unit; qSOFA, quick SOFA; SOFA, Sequential Organ Failure Assessment; WBC, white blood cell count; APACHE, Acute Physiology and Chronic Health Evaluation.

**Table 2:** Microorganisms isolated from blood cultures of patients with severe sepsis and septic shock.

Isolate	n (%)
Gram-positive bacteria	
Oxacillin-resistant *Staphylococcus aureus*	2 (5)
Oxacillin-sensitive *Staphylococcus aureus*	1 (2.5)
Other *Staphylococci*	5 (12.5)
*Enterococcus faecium*	4 (10)
*Streptococcus pneumoniae*	1 (2.5)
Unspecified Gram-positive *bacilli*	2 (5)
Gram-negative bacteria	
*Klebsiella pneumoniae*	5 (12.5)
*Escherichia coli*	4 (10)
*Aeromonas* species	2 (5)
*Pseudomonas aeruginosa*	2 (5)
*Proteus mirabilis*	2 (5)
*Chryseobacterium meningosepticum*	1 (2.5)
*Stenotrophomonas maltophilia*	1 (2.5)
*Morganella morganii*	1 (2.5)
*Myroides species*	1 (2.5)
*Fusobacterium* species	1 (2.5)
Fungus	
*Candida* species	5 (12.5)

### Comparison of kallistatin and other biomarkers related to in-hospital mortality, septic shock, positive blood culture, and ARDS

**Fig 2:**
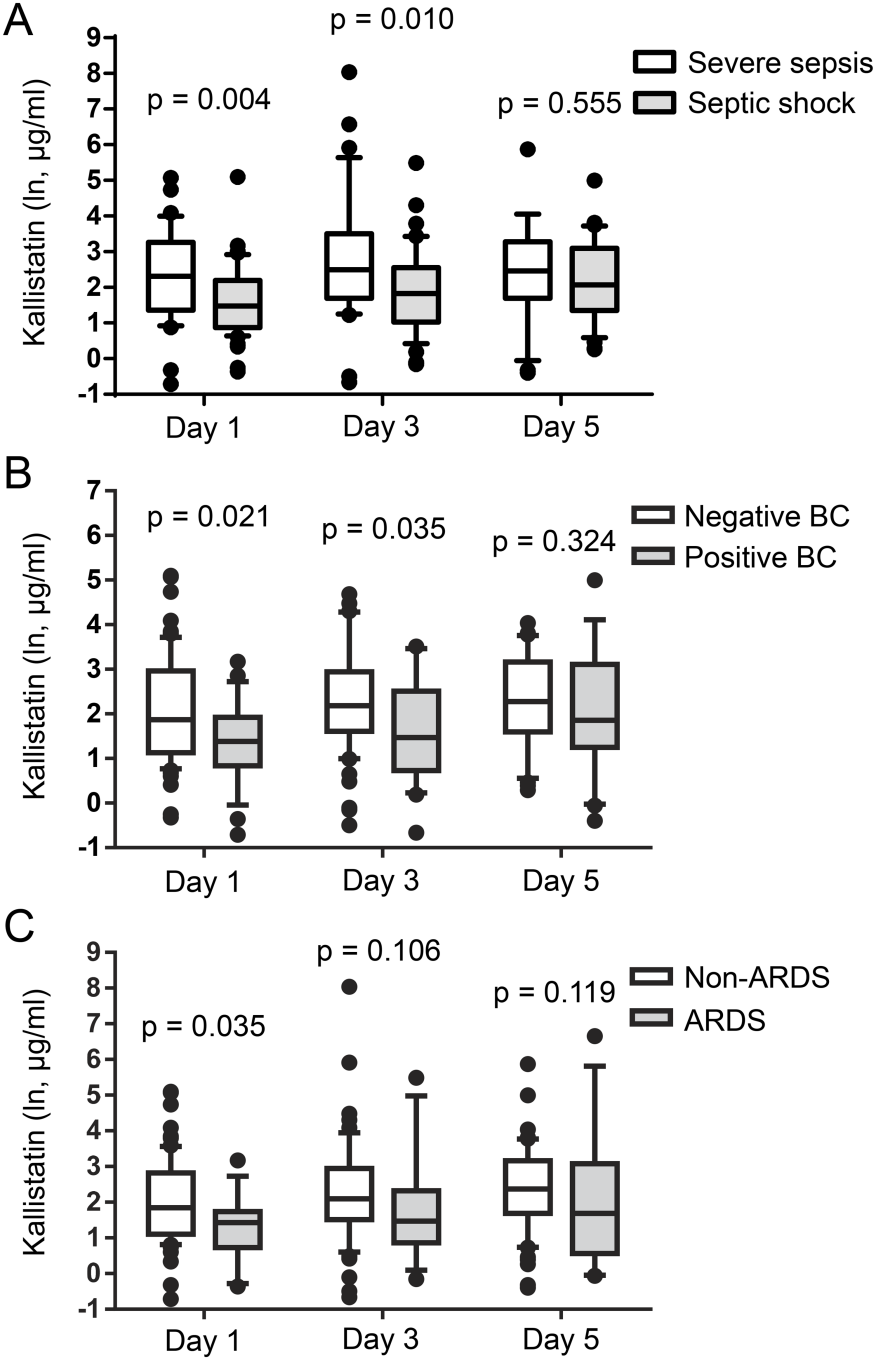
Plasma kallistatin levels on days 1, 3 and 5 of ICU admission in severe sepsis versus septic shock, positive blood culture versus negative blood culture, and ARDS versus non-ARDS. Plasma kallistatin levels on days 1, 3 and 5 were compared between patients with severe sepsis and septic shock (A), patients who were blood culture positive and blood culture negative (B), and ARDS and non-ARDS patients (C). The Mann-Whitney nonparametric *U*-test was used for comparisons between the groups. BC, blood culture.

Day 1 kallistatin levels were significantly lower in patients who died compared with those who survived (p = 0.031). In contrast, levels of IL-6 and IL-8 on days 1, 3, and 5 and day 1 C-reactive protein (CRP) were significantly higher in nonsurvivors compared with survivors. There was no significant difference in the levels of kallikrein, TNF-α, and IL-1β between survivors and nonsurvivors (Table 3). Day 1 and 3 kallistatin levels were also significantly lower in patients with septic shock compared with severe sepsis (day 1, p = 0.004 and day 3, p = 0.010) (Fig 2A) and in patients who had positive blood cultures versus negative blood cultures (day 1, p = 0.021 and day 3, p = 0.035) (Fig 2B). Moreover, day 1 kallistatin levels were significantly lower in patients who developed ARDS compared with those who did not (p = 0.035) (Fig 2C). In contrast, higher day 1 levels of TNF-α, IL-6, IL-8, and CRP (p = 0.013, p < 0.001, p = 0.001, and p = 0.017, respectively), but not IL-1β (p = 0.057), were observed in patients with septic shock compared with those with severe sepsis (S1 Table). CRP levels were significantly higher (p = 0.017) in patients who developed ARDS compared with non-ARDS patients. There was no significant difference in TNF-α, IL-1β, IL-6, and IL-8 levels between ARDS and non-ARDS patients (S1 Table). In addition, there was no significant difference in the levels of TNF-α, IL-1β, IL-6, IL-8, and CRP between patients with positive blood cultures and those with negative blood cultures (S1 Table).

**Table 3:** Comparisons of plasma biomarkers between survivors and nonsurvivors of severe sepsis and septic shock.

Variables*	Survivors (n = 57)	Nonsurvivors (n = 29)	p value
Kallistatin (μg/ml)			
Day 1	3.7 (2.3–6.9)	2.8 (1.6–3.7)	0.031
Day 3	4.1 (3.0–8.9)	3.7 (1.5–6.0)	0.082
Day 5	5.3 (3.4–10.2)	3.3 (1.5–8.3)	0.058
Kallikrein (pg/ml)			
Day 1	602.2 (332.5–1261.5)	655.1 (287.5–952.0)	0.625
Day 3	621.6 (244.6–1354.5)	410.7 (120.6–950.7)	0.233
Day 5	487.3 (179.6–1243.0)	436.1 (153.9–937.1)	0.478
TNF-α (pg/ml)			
Day 1	9.1 (2.7–52.1)	17.9 (3.5–124.5)	0.304
Day 3	5.0 (2.3–34.7)	9.2 (3.1–61.2)	0.446
Day 5	3.5 (2.1–46.8)	13.8 (2.8–76.2)	0.170
IL-1β (pg/ml)			
Day 1	0.5 (0.2–1.0)	0.6 (0.2–2.0)	0.376
Day 3	0.4 (0.1–0.8)	0.4 (0.1–1.6)	0.183
Day 5	0.3 (0.1–0.9)	0.7 (0.4–1.3)	0.052
IL-6 (pg/ml)			
Day 1	44.7 (12.4–105.4)	85.7 (21.8–939.3)	0.035
Day 3	33.7 (10.4–61.9)	65.0 (23.1–262.3)	0.007
Day 5	21.1 (3.1–36.0)	50.3 (24.6–116.7)	0.001
IL-8 (pg/ml)			
Day 1	24.8 (12.2–42.4)	50.6 (22.9–281.1)	0.003
Day 3	14.7 (6.9–36.8)	45.1 (17.8–111.2)	0.002
Day 5	15.5 (5.0–30.8)	41.9 (15.8–169.1)	0.001
CRP (μg/ml)			
Day 1	79.2 (38.5–192.5)	155.5 (71.6–259.0)	0.008

Data are expressed as the median (interquartile range). TNF-α, tumor necrosis factor-α; IL, interleukin; CRP, C-reactive protein.

*Samples of 4 patients (1 survivor and 3 nonsurvivors) from day 3, and 12 (5 survivors and 7 nonsurvivors) from day 5 were missing.

### Relationship between kallistatin and other biomarkers on day 1 of ICU admission

There were inverse correlations between day 1 kallistatin and day 1 CRP (ρ = −0.336, p = 0.003), TNF-α (ρ = −0.263, p = 0.014), IL-1β (ρ = −0.231, p = 0.032), IL-6 (ρ = −0.320, p = 0.003), and IL-8 (ρ = −0.212, p = 0.051) levels.

### Association between kallistatin, 60-day mortality, and disease severity

**Fig 3:**
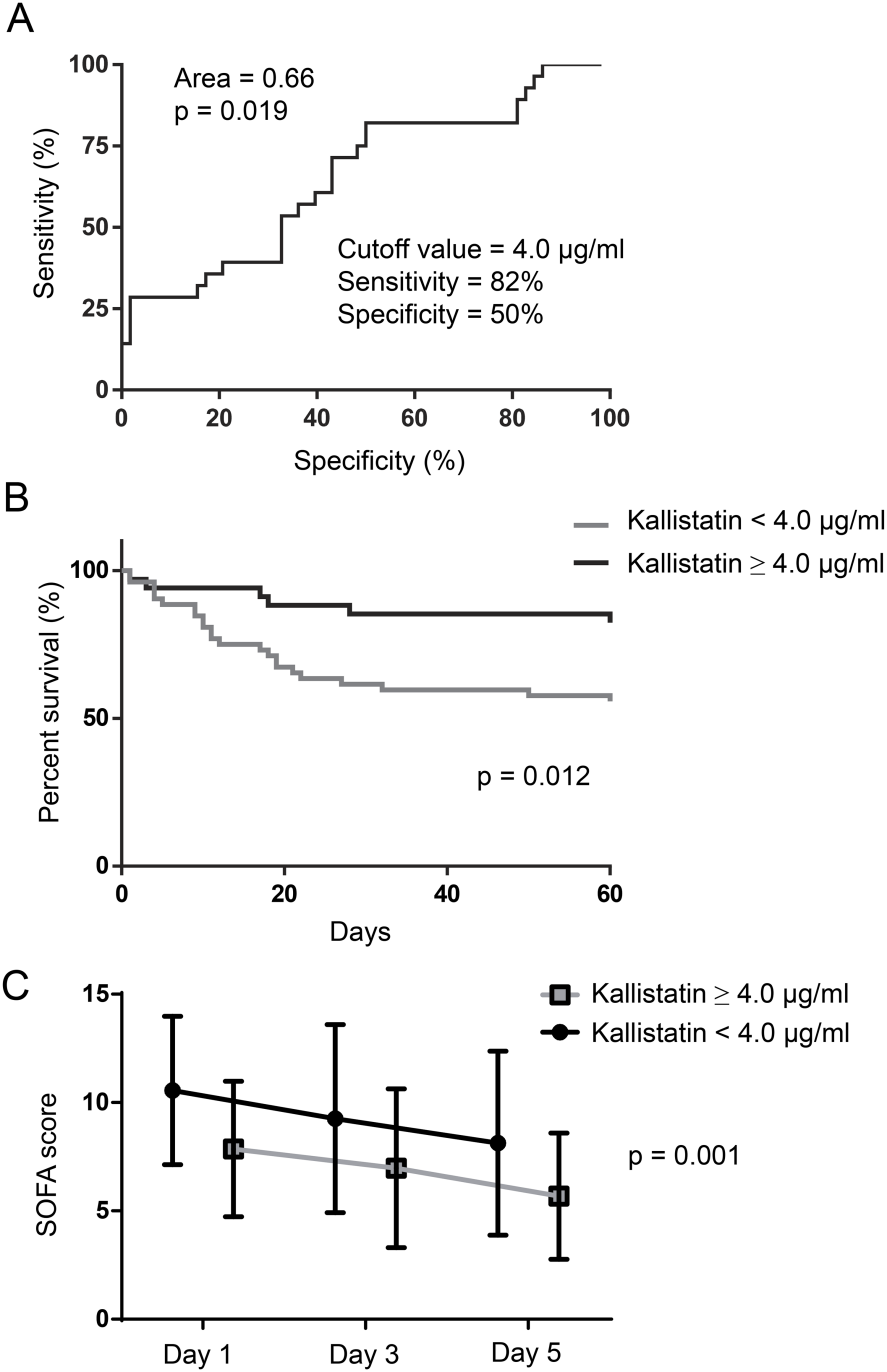
ROC curve and Kaplan-Meier curve analyses for 60-day mortality and stratified SOFA scores for days 1–5 days related to plasma kallistatin levels on day 1 of ICU admission in severe sepsis and septic shock patients. (A) ROC curves determining the cutoff value of day 1 kallistatin (4.0 μg/ml) to discriminate between survivors and nonsurvivors. (B) Kaplan-Meier curves of 60-day survival with patients grouped according to day 1 kallistatin levels ≥ 4.0 μg/ml or < 4.0 μg/ml. Log-rank test for comparisons between groups. (C) SOFA scores on days 1, 3, and 5 in patients grouped according to day 1 kallistatin levels ≥ 4.0 μg/ml or < 4.0 μg/ml. Mixed model comparisons between groups.

To determine the optimal cutoff values of day 1 plasma kallistatin for 60-day mortality, ROC curves were constructed with an AUC of 0.66 (p = 0.019) (Fig 3A). The optimum cutoff value was 4 μg/ml, corresponding to the maximum sum of sensitivity (82%) and specificity (50%) (Fig 3A). The diagnostic accuracy of other biomarkers and SOFA scores to predict 60-day mortality was also evaluated. The AUC for the ROC was higher for day 1 SOFA score (AUC = 0.78, p < 0.001) compared with kallistatin and the other biomarkers. However, the AUC improved when kallistatin and the other biomarkers were combined (AUC = 0.80, p < 0.001) (Table 4). The 60-day survival rate was further assessed by Kaplan-Meier analysis according to the determined cutoff value. Patients with high levels of plasma kallistatin (≥ 4 μg/ml) on day 1 had a better 60-day survival (p = 0.012) (Fig 3B). Furthermore, day 1 kallistatin levels were negatively correlated with day 1 SOFA score (ρ = −0.317, p = 0.003). Using the optimum cutoff value, high levels of plasma kallistatin were associated with lower SOFA scores over the first 5-day period in the ICU (Fig 3C).

**Table 4:** ROC curve analysis for 60-day mortality prediction in severe sepsis and septic shock patients.

Markers	Cutoff value	Sensitivity	Specificity	AUC (95% CI)	p value
Kallistatin	4.0	0.82	0.50	0.66 (0.53–0.78)	0.019
IL-6	161.8	0.46	0.86	0.65 (0.52–0.78)	0.026
IL-8	42.3	0.68	0.76	0.73 (0.62–0.84)	0.001
CRP	136.5	0.68	0.62	0.68 (0.55–0.80)	0.008
SOFA score	9.5	0.79	0.66	0.78 (0.67–0.89)	<0.001
Kallistatin + IL-6	–	–	–	0.73 (0.62–0.85)	0.001
Kallistatin + IL-8	–	–	–	0.78 (0.67–0.88)	<0.001
Kallistatin + CRP	–	–	–	0.70 (0.58–0.81)	0.030
Kallistatin + IL-6 + CRP	–	–	–	0.76 (0.65–0.86)	<0.001
Kallistatin + IL-8 + CRP	–	–	–	0.79 (0.69–0.89)	<0.001
Kallistatin + IL-6 + IL-8	–	–	–	0.80 (0.70–0.90)	<0.001
Kallistatin + IL-6 + IL-8 + CRP	–	–	–	0.80 (0.70–0.90)	<0.001

ROC, receiver operating characteristic; AUC, area under the ROC curve; CI, confidence interval; IL, interleukin; CRP, C-reactive protein; SOFA, Sequential Organ Failure Assessment.

### Univariate and multivariable analysis of baseline severity scores and biomarkers

Univariate Cox regression modeling showed that high day 1 kallistatin levels (≥ 4 μg/ml) were associated with a significantly lower risk of 60-day death (HR 0.28 (95% CI, 0.11–0.74), p = 0.010) (S2 Table). Other factors including septic shock, ARDS, day 1 levels of IL-1β, IL-6, IL-8 and CRP, and day 1 SOFA and APACHE II scores, were also predictive of 60-day mortality. However, when adjusted for these factors with p < 0.05 in univariate analysis, the association between high day 1 kallistatin level and 60-day mortality was not significant (HR 0.72 (95% CI, 0.24–2.17), p = 0.558) (S2 Table). To determine whether high day 1 kallistatin level was independently associated with septic shock, positive blood culture, and the risk of ARDS, a backward multivariable logistic regression analyses was performed. Only high kallistatin levels were associated with a significantly lower risk of septic shock, ARDS, and positive blood culture (OR 0.29 (95% CI, 0.12–0.73), p = 0.008 for septic shock; OR 0.17 (95% CI, 0.04–0.80), p = 0.025 for ARDS; OR 0.33 (95% CI, 0.11–0.99), p = 0.047 for positive blood culture) (S3 Table).

## Discussion

This is the first study to investigate the plasma concentrations of kallistatin in patients with severe sepsis and septic shock in an ICU. We found that the plasma kallistatin levels at ICU admission were lower in patients with septic shock compared with those with severe sepsis, and lower kallistatin levels were associated with increased risk of death, the development of ARDS, and a higher rate of positive blood culture. Kallistatin levels were inversely correlated with SOFA scores, and low day 1 kallistatin levels (< 4 μg/ml) were related to elevated SOFA scores over the first 5 days after ICU admission. The hospital mortality rate of 34% in this study is similar with that reported previously [22].

Kallistatin is mainly expressed in the liver, but is also widely distributed in the pancreas, kidney, lung, heart, colon, and blood vessels [23]. Human kallistatin levels are significantly decreased in liver cirrhosis, sepsis, and inflammatory bowel disease, suggesting kallistatin is consumed in these diseases [5, 24, 25]. We previously demonstrated that patients with severe pneumonia had decreased plasma levels of kallistatin [20]. Plasma kallistatin levels in normal subjects were reported as 22.1± 3.5 μg/ml [5]. We reported a median kallistatin level of 17.2 μg/ml (5.3–82.7 μg/ml) in normal subjects using the same assay as this study [20]. Consistent with these studies investigating patients with sepsis or pneumonia [5, 20], we revealed significantly decreased kallistatin levels in critically ill patients with severe sepsis and septic shock.

Serial severity-score systems are well-established markers of outcome in critically ill patients with sepsis [22]. Consistently, we found that patients who survived during hospitalization had lower APACHE II and SOFA scores compared with those who died and day 1 SOFA score was an independent risk factor for 60-day mortality. Many inflammatory mediators are used as biomarkers to predict patient outcomes because sepsis upregulates acute inflammatory processes [26, 27]. In this study, we also observed higher plasma levels of IL-6, IL-8, and CRP in nonsurvivors compared with survivors. Consistent with our previous study in patients with severe CAP, we observed lower kallistatin levels in nonsurvivors compared with survivors. Kaplan-Meier survival-curve analysis revealed a better survival rate in patients with high kallistatin levels than in those with low levels. However, none of these biomarkers was significantly associated with 60-day mortality in the multivariable analysis. This might reflect too few enrolled patients to demonstrate a difference in a heterogeneous population with sepsis. Kallikrein was reported to be involved in the pathogenesis of sepsis [28], but we found no difference in kallikrein levels between survivors and nonsurvivors, suggesting effects on the outcomes of sepsis are not related to interactions with kallikrein.

Of note, the ROC—AUC of kallistatin for 60-day mortality was low but comparable to our previous study investigating kallistatin in CAP patients [20]. However, we found that the AUC improved when the inflammatory markers were combined. This might indicate that kallistatin in combination with other inflammatory markers might identify patients with a high risk of death to refine risk stratification. In particular, high kallistatin level was independently associated with septic shock, risk of ARDS, and positive blood culture, whereas other inflammatory markers were not. Therefore, kallistatin might be used to identify high-risk patients who develop a complicated clinical course and require aggressive intervention, while directing a conservative approach to low-risk patients.

Several animal studies using various sepsis models have shown that kallistatin is protective against organ injury and mortality. Using a mouse model of streptococcal infection, we previously demonstrated that kallistatin gene transfer reduced bacteremia, skin and liver damage, and mortality, as well as decreased inflammatory cell numbers and inflammatory cytokine levels at local infection sites [14]. In a mouse model of LPS-induced acute lung injury, kallistatin gene or protein delivery attenuated lung inflammation, apoptosis, and injury, as well as increased survival [17]. Li *et al*., used mouse models of polymicrobial sepsis and endotoxemia to show that treatment with kallistatin prior to or after the onset of sepsis attenuated inflammation, multiorgan injury, and peritoneal bacterial counts, as well as improved survival [15, 16]. In keeping with these findings, we observed that circulating kallistatin levels were inversely correlated with SOFA scores, an indicator of the extent of organ injury. Patients with high day 1 kallistatin levels (≥ 4 μg/ml) were associated with decreased mortality and disease severity over the first 5 days in the ICU. This might suggest that kallistatin can be used for the simple assessment of disease severity until SOFA can be calculated or in cases where SOFA cannot be calculated because of missing values. The biological functions of kallistatin may contribute to increased survival, lower bacteremia rate, and reduced risk of ARDS and septic shock in patients with high levels of kallistatin. Likewise, consistent with our previous study of patients with severe CAP [20], the anti-inflammatory effect of kallistatin may explain the observations that plasma kallistatin levels were negatively correlated with inflammatory cytokines/chemokines. CRP has long been recognized as an acute-phase reactant [29]; in contrast, kallistatin has been observed to be a negative acute-phase protein in patients with severe infection. Our results imply a potential role for kallistatin in the severity and outcome prognosis in patients with severe sepsis and septic shock.

The main strength of this study was the serial measurement of kallistatin and biomarkers involved in the pathogenesis of sepsis simultaneously to evaluate the relationship between kallistatin, these biomarkers, and patient outcomes. There were some limitations in our study. First, its small sample size in a single medical center prevented subgroup analysis to examine differences in the effects of kallistatin on clinical outcomes according to comorbidities or site of infection. Second, we did not record data regarding specific therapeutic interventions, which may affect the results through unknown interactions with kallistatin, such as corticosteroids used in patients with septic shock. However, we collected initial blood samples at ICU admission when corticosteroids had not been administered for the majority of our patients. Third, we did not assess the long-term study outcomes after hospital discharge. Further study is needed to determine whether there are long-term effects of kallistatin on the study outcomes.

## Conclusions

The plasma levels of kallistatin in patients with septic shock were significantly lower compared with severe sepsis patients, and patients with a lower level of kallistatin had more severe illness, increased risk of death, and higher rate of positive blood culture and were more likely to develop ARDS. However, kallistatin was not an independent predictor of 60-day mortality.

## Supporting information

S1 Data The dataset of all enrolled patients. (XLSX)

S1 Table Comparisons of day 1 plasma biomarkers between severe sepsis and septic shock, ARDS and non-ARDS, and positive blood culture and negative blood culture. (DOCX)

S2 Table Cox univariate and multivariable analysis for 60-day mortality in patients with severe sepsis and septic shock. (DOCX)

S3 Table Univariate and multivariable analyses for factors independently associated with septic shock, ARDS, and positive blood culture in patients with severe sepsis and septic shock. (DOCX)
