# Supplementary material for: Plasma kallistatin in critically ill patients with severe sepsis and septic shock
Source: PLoS One. 2017 May 24;12(5):e0178387. doi: 10.1371/journal.pone.0178387 (PMC5443576; doi:10.1371/journal.pone.0178387)
Supplement: S1 Table — (DOCX) [file pone.0178387.s002.docx]

**S1 Table. Comparisons of day 1 plasma biomarkers between severe sepsis and septic shock, ARDS and non-ARDS, and positive blood culture and negative blood culture.**

|  | **Septic shock** | |  | **ARDS** | |  | **Blood culture** | |  |
| --- | --- | --- | --- | --- | --- | --- | --- | --- | --- |
| **Variables** | **No** | **Yes** | **p value** | **No** | **Yes** | **p value** | **Negative** | **Positive** | **p value** |
| Kallistatin (μg/ml) | 5.0 (2.6–9.6) | 2.8 (1.8–4.6) | 0.004 | 3.6 (2.2–7.1) | 2.7 (1.7–3.3) | 0.035 | 3.7 (2.2–7.8) | 2.6 (1.8–3.8) | 0.021 |
| Kallikrein (pg/ml) | 565.3 (267.5–1279.1) | 635.6 (399.5–910.8) | 0.780 | 645.4 (307.2–1247.8) | 480.0 (400.7–848.0) | 0.594 | 537.3 (305.3–1234) | 680.5 (432.3–947.8) | 0.714 |
| TNF-α (pg/ml) | 4.1 (2.0–17.3) | 26.2 (4.5–109.7) | 0.013 | 12.8 (2.9–74.7) | 8.8 (3.7–65.5) | 0.773 | 9.2 (2.8–116.4) | 14.6 (4.1–36.2) | 0.762 |
| IL-1β (pg/ml) | 0.5 (0.1–0.9) | 0.6 (0.3–1.6) | 0.057 | 0.5 (0.2–1.0) | 1.0 (0.2–2.3) | 0.071 | 0.5 (0.2–1.3) | 0.7 (0.2–0.9) | 0.581 |
| IL-6 (pg/ml) | 26.2 (5.8–57.0) | 80.5 (30.6–570.9) | <0.001 | 43.4 (12.8–136.4) | 107.3 (33.5–572.9) | 0.067 | 55.3 (19.0–153.6) | 42.2 (12.3–170.0) | 0.650 |
| IL-8 (pg/ml) | 19.1 (4.4–38.5) | 39.6 (20.9–129.4) | 0.001 | 29.1 (12.6 –79.4) | 47.3 (20.2–149.8) | 0.088 | 27.6 (13.1–70.8) | 39.6 (20.4–143.5) | 0.258 |
| CRP (μg/ml) | 72.3 (30.2–170.9) | 155.5 (52.7–214.1) | 0.017 | 98.6 (39.8–192.4) | 198.7 (91.3–232.2) | 0.017 | 115.8 (43.7–192.7) | 182.2 (46.2–222.2) | 0.262 |

Data are expressed as the median (interquartile range). ARDS, acute respiratory distress syndrome; TNF-α, tumor necrosis factor-α; IL, interleukin; CRP, C-reactive protein.
