# Supplementary material for: Plasma kallistatin in critically ill patients with severe sepsis and septic shock
Source: PLoS One. 2017 May 24;12(5):e0178387. doi: 10.1371/journal.pone.0178387 (PMC5443576; doi:10.1371/journal.pone.0178387)
Supplement: S2 Table — (DOCX) [file pone.0178387.s003.docx]

**S2 Table. Cox univariate and multivariable analysis for 60-day mortality in patients with severe sepsis and septic shock.**

| **Variables** | **p value** | **HR** | **95%CI** |
| --- | --- | --- | --- |
| Univariate analysis |  |  |  |
| Age | 0.256 | 1.02 | 0.99–1.04 |
| Sex (male) | 0.449 | 0.75 | 0.35–1.59 |
| Septic shock | 0.013 | 3.40 | 1.29–8.95 |
| ARDS | 0.027 | 2.45 | 1.11–5.42 |
| Kallistatin > 4 μg/ml | 0.010 | 0.28 | 0.11–0.74 |
| Kallikrein | 0.510 | 1.00 | 0.99–1.00 |
| TNF-α | 0.403 | 1.00 | 0.99–1.00 |
| IL-1β | <0.001 | 1.13 | 1.07–1.20 |
| IL-6 | 0.002 | 1.00 | 1.00–1.00 |
| IL-8 | 0.020 | 1.00 | 1.00–1.00 |
| CRP | 0.001 | 1.01 | 1.00–1.01 |
| SOFA score | <0.001 | 1.33 | 1.19–1.50 |
| APACHE II score | <0.001 | 1.12 | 1.06–1.19 |
| Multivariable analysis |  |  |  |
| Septic shock | 0.950 | 0.97 | 0.32–2.89 |
| ARDS | 0.825 | 0.89 | 0.32–2.48 |
| Kallistatin > 4 μg/ml | 0.558 | 0.72 | 0.24–2.17 |
| IL-1β | 0.057 | 1.11 | 1.00–1.23 |
| IL-6 | 0.589 | 1.00 | 1.00–1.00 |
| IL-8 | 0.251 | 1.00 | 1.00–1.00 |
| CRP | 0.052 | 1.00 | 1.00–1.01 |
| SOFA score | 0.023 | 1.24 | 1.03–1.50 |
| APACHE II score | 0.437 | 1.04 | 0.95–1.14 |

HR, hazard ratio; CI, confidence interval; ARDS, acute respiratory distress syndrome; TNF-α, tumor necrosis factor-α; IL, interleukin; CRP, C-reactive protein; SOFA, Sequential Organ Failure Assessment; APACHE II, Acute Physiology and Chronic Health Evaluation II.
