# Supplementary material for: Plasma kallistatin in critically ill patients with severe sepsis and septic shock
Source: PLoS One. 2017 May 24;12(5):e0178387. doi: 10.1371/journal.pone.0178387 (PMC5443576; doi:10.1371/journal.pone.0178387)
Supplement: S3 Table — (DOCX) [file pone.0178387.s004.docx]

**S3 Table. Univariate and multivariable analyses for factors independently associated with septic shock, ARDS, and positive blood culture in patients with severe sepsis and septic shock.**

|  | **Septic shock** | | | | **ARDS** | | | | **Blood culture** | | | |
| --- | --- | --- | --- | --- | --- | --- | --- | --- | --- | --- | --- | --- |
|  | **Univariate** | | **Multivariable** | | **Univariate** | | **Multivariable** | | **Univariate** | | **Multivariable** | |
| **Variables** | **OR (95%CI)** | **p** | **OR (95%CI)** | **p** | **OR (95%CI)** | **p** | **OR (95%CI)** | **p** | **OR (95%CI)** | **p** | **OR (95%CI)** | **p** |
| Age | 1.00 (0.97–1.03) | 0.994 | – | – | 1.01 (0.97–1.05) | 0.582 | – | – | 0.99 (0.96–1.02) | 0.497 | – | – |
| Gender (male) | 1.50 (0.60–3.74) | 0.381 | – | – | 0.82 (0.26–2.52) | 0.723 | – | – | 1.63 (0.56–4.72) | 0.368 | – | – |
| Kallistatin > 4 μg/ml | 0.29 (0.12–0.73) | 0.008 | 0.29 (0.12–0.73) | 0.008 | 0.17 (0.04–0.80) | 0.025 | 0.17 (0.04–0.80) | 0.025 | 0.33 (0.11–0.99) | 0.047 | 0.33 (0.11–0.99) | 0.047 |
| Kallikrein | 1.00 (0.99–1.00) | 0.818 | – | – | 1.00 (0.99–1.00) | 0.416 | – | – | 1.00 (0.99–1.00) | 0.725 | – | – |
| TNF-α | 1.00 (0.99–1.00) | 0.717 | – | – | 1.00 (1.00–1.00) | 0.624 | – | – | 1.00 (0.99–1.00) | 0.339 | – | – |
| IL-1β | 1.64 (0.87–3.11) | 0.127 | – | – | 1.06 (0.96–1.17) | 0.274 | – | – | 0.80 (0.53–1.20) | 0.278 | – | – |
| IL-6 | 1.00 (1.00–1.00) | 0.144 | – | – | 1.00 (1.00–1.00) | 0.144 | – | – | 1.00 (0.99–1.00) | 0.466 | – | – |
| IL-8 | 1.00 (0.99–-1.00) | 0.243 | – | – | 1.00 (1.00–1.00) | 0.567 | – | – | 1.00 (1.00–1.00) | 0.541 | – | – |
| CRP | 1.01 (1.00–1.01) | 0.019 | – | – | 1.01 (1.00–1.01) | 0.013 | – | – | 1.00 (0.99–1.00) | 0.340 | – | – |
| SOFA score* | – | – | – | – | – | – | – | – | 1.09 (0.95–1.25) | 0.200 | – | – |
| APACHE II score* | – | – | – | – | – | – | – | – | 1.01 (0.95-1.08) | 0.692 | – | – |

ARDS, acute respiratory distress syndrome; OR, odds ratio; CI, confidence interval; TNF-α, tumor necrosis factor-α; IL, interleukin; CRP, C-reactive protein; SOFA, Sequential Organ Failure Assessment; APACHE II, Acute Physiology and Chronic Health Evaluation II. *The variables SOFA and APACHE II were not included in logistic regression analyses for septic shock and ARDS because shock and hypoxemia are the constituents of these severity-score systems.
